# Worm corpses affect quantification of dauer recovery

**DOI:** 10.17912/micropub.biology.000121

**Published:** 2019-06-11

**Authors:** Carolaing Gabaldon, Andrea Calixto

**Affiliations:** 1 Centro de Genómica y Bioinformática, Facultad de Ciencias, Universidad Mayor, Santiago de Chile, Chile; 2 Centro Interdisciplinario de Neurociencias de Valparaíso, Facultad de Ciencias, Universidad de Valparaíso, Valparaíso, Chile

**Figure 1.**
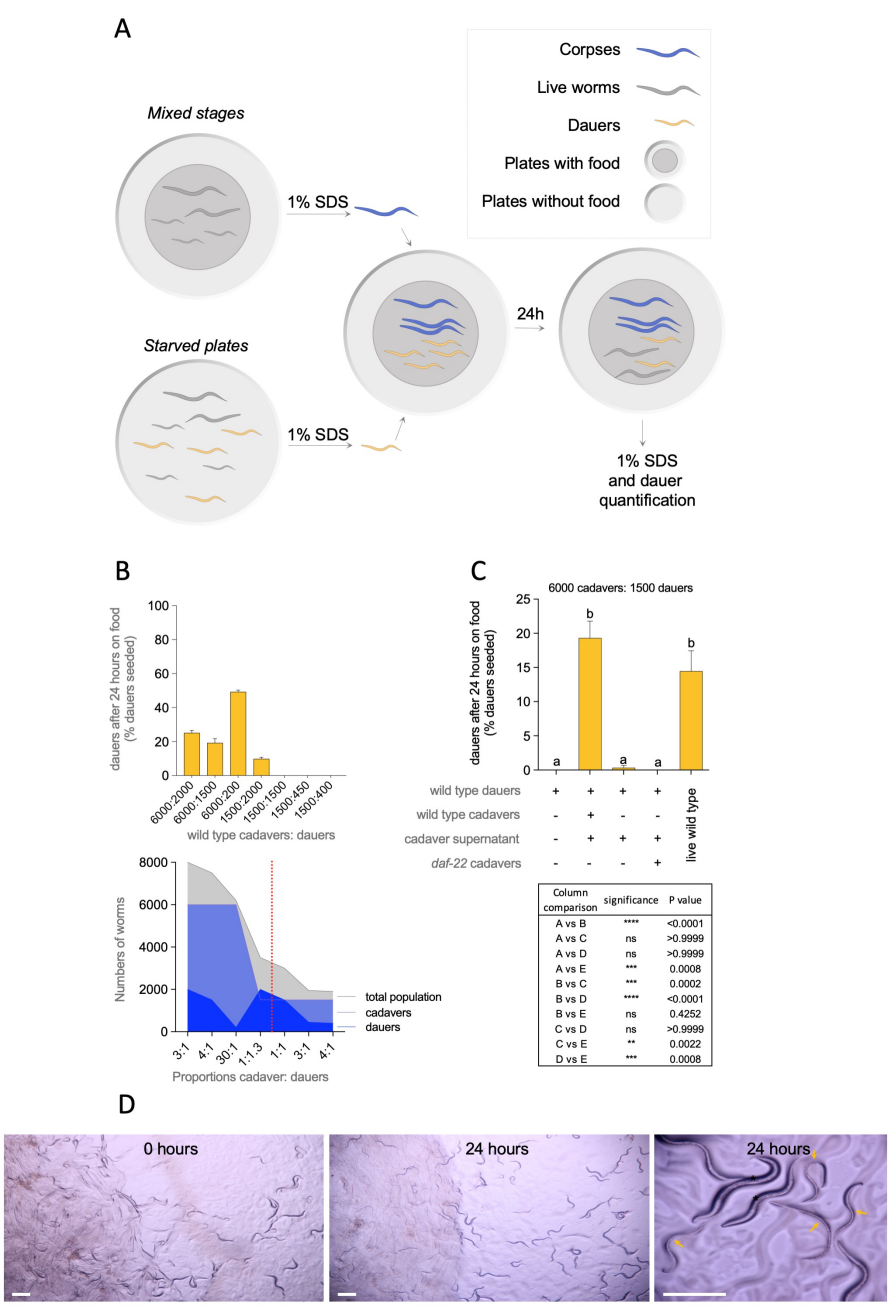
Dauer recovery on *E. coli* OP50 under different conditions of population size and genotype. (A) Methodological outline used for recovery of dauers in the presence of corpses or live animals in plates with *E. coli* OP50 food. (B) Percent of dauer recovery after 24 hours of exposure to food on different proportions of cadavers and dauers (upper panel) and representation of numbers of dauers, cadavers and total population on each proportion (lower panel). (C) Percent of dauer recovery after 24 hours of exposure to food in the proportion 1:4 (6000 cadavers to 1500 dauers) on different conditions of wild type and *daf-22* genetic backgrounds. *a* and *b* represent statistically different conditions. All comparisons, significance and P value are indicated. (D) Representative pictures of plates on the first day of dauer exposure to *E. coli* OP50 in the presence of corpses (0 hours) and 24 hours after. The third photograph is a zoom of photographs at 24 hours to show animals that exited dauer (stars) and dauers (arrows). Bars are 200 mm. Photographs were taken with a Cannon camera EOS Rebel T3i on a dissecting Nikon AZ100 in 2X objective at 1/400s exposure time, with ISO 100, focal distance 50 mm and 5X or 8X magnification.

## Description

Diapause entry and recovery are behavioral adaptations that ensure the survival to environmental changes of animals capable of this type of developmental arrest. *Caenorhabditis elegans* enters diapause by forming the dauer larvae and at recovery resume post dauer L3 fate (Karp and Ambros, 2012). The precise quantification of dauer exit is required to estimate: post-dauer life span (Klass and Hirsh, 1976); dauer recovery ability after food replenishment (Cassada and Russell, 1975); to investigate components of bacterial extracts that promote dauer exit (Mylenko et al., 2016); the effect of enzymes and proteins involved in dauer recovery (Reape and Burnell, 1990), metabolic analysis at dauer recovery (Houthoofd et al., 2002), inhibition of dauer recovery by pheromone (Golden and Riddle, 1984a, Golden and Riddle, 1984b); global analysis of dauer gene expression (Wang and Kim, 2003) or longevity of dauers (Caneo et al., 2019). Dauers can be discriminated from non-dauers because they are resistant to 1% SDS treatment (Cassada and Russell, 1975). This method is also used to place the resulting dauers in food plates to quantify dauer recovery. We serendipitously found that when dauers alongside with corpses resulting from 1% SDS treatment were seeded in *E. coli* OP50 plates, a significant number of animals remained in diapause after 24 hours of food exposure. Three factors could be affecting dauer recovery: population size, ratio between dauer and cadaver, and the spatial distribution of them. To investigate how corpses affect dauer recovery, we mixed specific numbers of dauers (200, 400, 450 or 1500) and cadavers (1500 or 6000) and quantified dauer recovery after 24 hours (Figure 1A). Dauers and cadavers were well mixed to minimize the influence of spatial distribution of the worms. Worm cadavers affected recovery from dauers in a quorum-dependent fashion as observed before for live animals (Golden and Riddle, 1984a). Cadavers totaling 6000 in a proportion of 4:1, 3:1 or 30:1 (cadaver:dauer) prevented between 20-50% dauer recovery in plates with plenty of food. Cadavers totaling 1500 in a proportion of 4:1, 3:1, or 1:1 did not have the same effect on dauer recovery. However, when dauers outnumbered 1500 corpses in a proportion 1:1.3 there was dauer retention (Figure 1B). Figure 1B (bottom) shows that total number of animals (corpses and dauers) is also an important factor in dauer recovery. The proportion 4:1 and 3:1 were not sufficient by themselves. In our experiments, the minimum total number of worms for dauer retention was 3500. Using a 4:1 proportion (6000:1500), we tested whether dauer recovery impairment was dependent on pheromone from cadavers by using *daf-22*mutants of pheromone synthesis (Golden and Riddle, 1985). *daf-22* cadavers did not prevent dauer exit in the 4:1 proportion, suggesting that pheromones are retained in corpses and are not sensed by dauers (Figure 1C). Importantly, supernatant from cadavers was not sufficient to induce dauer retention, suggesting that cadavers themselves impair dauer recovery. As expected, 6000 live animals prevented dauer recovery in the presence of food (Figure 1C). Our experiments show that cadavers retain the ability to be sensed by dauers as live worms and constitute a confounding factor in dauer recovery quantification. Dauer recovery is best achieved when only dauer larvae are placed on plates*.*

## Reagents

*Strains:* We used wild type (N2) and DR476 *[daf-22 (m130)],* which were obtained from the CGC.

*Dauer isolation*: Dauers were obtained from 7-day starved plates initially fed with *E. coli* OP50. Plates were washed with 1 mL of M9 and transferred to an Eppendorf tube. The pellet was treated with 1 mL of 1% SDS for 20 minutes. The tube was centrifuged for 2 min at 2500 rpm, the supernatant eliminated and the dauer and corpses pellet seeded on a plate without bacteria. To further isolate dauers from worm carcasses, dauers were allowed to crawl off the pellet for 30 to 60 minutes (Caneo et al., 2019). The portion of the plate with the remaining pellet was excised from the plate with a scalpel and dauers collected with M9 and quantified.

*Dauer recovery:* For the recovery of dauers, 60 mm NGM plates were seeded with *Escherichia coli* (OP50). Bacteria was grown in LB medium for 6 hours until the culture reached an OD of 1. 400 ml of the bacterial culture was added to the plates and allowed to dry for 24 hours at room temperature to subsequently be stored at 4°C or used.

*Preparation of corpses:* Corpses were obtained from mixed plates with high density of worms with an approximate count of 5000 total worms. As a general rule after examining many plates, the proportion of animals in L1-2, L3, L4 and adults was relatively constant: 42% of animals treated were L1-L2, 24% were L3, 16% were L4 and 17% were adults. Animals from a mixed stage plate were washed once with M9 and treated with 1% SDS for 20 min. After SDS treatment tubes were centrifuged for 2 minutes at 2500 rpm. Supernatant was discarded, leaving 45 ml of liquid remaining. To count the number of corpses samples treated with 1% SDS were diluted 1:10 in M9. 10 μl of each dilution was used to count the number of corpses under a Nikon SMZ745 stereoscope.

The mixture of cadavers and dauers was made in a tube (after having the quantification of each one), mixed by pipetting and seeded on a 60 mm plate. Mixed cadavers and dauers were always seeded together in one spot over the *E. coli* OP50 food lawn.

*Experimental replicas and statistics:* All experiments were done at least 3 times (three biological replicas, started in different days and from different starting plates). Each biological replica contained a triplicate (three technical replicas). Statistical analysis was done by a one-way ANOVA with post-hoc analysis (Tukey’s test).
